# Falling Rates of Malaria among U.S. Military Service Members in Afghanistan Substantiate Findings of High Compliance with Daily Chemoprophylaxis

**DOI:** 10.4269/ajtmh.2012.12-0277a

**Published:** 2012-11-07

**Authors:** Remington L. Nevin

**Affiliations:** Department of Preventive Medicine; Bayne-Jones Army Community Hospital; Fort Polk, Louisiana; E-mail: remington.nevin@us.army.mil

Dear Sir:

I read with interest the report by Brisson and Brisson that showed higher compliance among U.S. military service members in Afghanistan with daily as compared with weekly malaria chemoprophylaxis.^1^ By conventional wisdom these results appear counterintuitive, and they stand in contrast to earlier findings among civilian travelers^2^ and military personnel^3^ that suggest observed compliance should be higher with weekly dosing. Despite this, their findings of high compliance are substantiated by recent evidence of ecological association among U.S. military personnel in Afghanistan between falling rates of malaria and changes in chemoprophylaxis policy that now favor daily medications.

Although weekly mefloquine was previously the drug of choice in Afghanistan,^4^ in early 2009 the U.S. Army prohibited the widespread use of weekly mefloquine and adopted daily doxycycline as its preferred chemoprophylaxis, recognizing that mefloquine had been routinely misprescribed to soldiers with mental health contraindications.^5^ Atovaquone/proguanil (marketed in the United States as Malarone) also became more widely available.^6^

From 2006 through 2008, before this policy change, the U.S. military reported 179 total cases of malaria among troops deployed to Afghanistan.^7^ After this policy change, from 2009 through 2011, the reported number fell to only 170 total malaria cases over an equivalent period.^8^–^10^ This drop is particularly significant given that the total deployed troop strength more than tripled between these periods, from 78,500 to 257,800, according to recent cumulative mid-year estimates from the Brookings Institution.^11^

In other words, among U.S. military personnel in Afghanistan, over these past three years, and co-incident with the shift away from mefloquine as a preferred chemoprophylaxis, the rate of malaria has actually fallen over 70%. Today in Afghanistan, malaria affects only about 1 U.S. service member per 1,000 per year^10^; this rate is extremely low by historical standards, particularly as compared with rates during prior operations in areas of comparable endemicity, including Somalia,^12^ where they were nearly ten times higher.^13^

It seems more than coincidence that a significant drop in malaria rates accompanied the switch away from weekly mefloquine and toward daily medications. As the data of Brisson and Brisson suggest, and notwithstanding previous considerations that weekly dosing might improve compliance,^14^ today's soldiers may be less likely to adhere to mefloquine chemoprophylaxis because of growing awareness of its strong association with psychiatric symptoms and its potential to induce neurotoxicity.^15^

Senior U.S. military medical officials now acknowledge that the neuropsychiatric side effects caused by mefloquine may confound the diagnosis and management of post-traumatic stress disorder and traumatic brain injury, making its routine use less desirable.^16^ In recognition of emerging consensus that mefloquine may be poorly suited for continued widespread use among U.S. combat troops,^17^ recent policy changes in Afghanistan now emphasize daily Malarone as the preferred chemoprophylaxis when doxycycline is contraindicated.^18^ Although much higher in cost relative to other medications, the cost of even this most expensive medication comprises only a small fraction of the total costs of a year-long deployment to Afghanistan, which by recent estimates may be as high as $500,000^19^ to $1,000,000^20^ annually.
